# Supplementary material for: High‐throughput discovery of functional disordered regions: investigation of transactivation domains
Source: Mol Syst Biol. 2018 May 14;14(5):e8190. doi: 10.15252/msb.20188190 (PMC5949888; doi:10.15252/msb.20188190)
Supplement: Supplementary file 1 — Appendix [file MSB-14-e8190-s001.pdf]

High-throughput discovery of functional disordered protein regions: investigation of transactivation domains

Charles N. J. Ravarani[1,*], Tamara Y. Erkina[2], Greet De Baets[1], Daniel C. Dudman[2], Alexandre M. Erkine[2,*], M. Madan Babu[1,*]

[1]*MRC Laboratory of Molecular Biology, Francis Crick Avenue, Cambridge CB2 0QH, UK*
[2]*Butler University, 4600 Sunset Avenue, Indianapolis, IN 46208, USA*

Corresponding authors: ravarani@mrc-lmb.cam.ac.uk, aerkine@butler.edu, madanm@mrc-lmb.cam.ac.uk

TABLE OF CONTENTS FOR APPENDIX FIGURES S1-S13

Appendix Figure S1. Details of the experimental design	1
Appendix Figure S2. Flowchart of the next generation sequencing data processing pipeline and Machine Learning	2
Appendix Figure S3. Machine learning procedure flowchart	3
Appendix Figure S4. Spot dilution assay for sequences identified during the screen of the random library	4
Appendix Figure S5. Performance of classical features associated with TAD using logistic regression models	5
Appendix Figure S6. Performance of the combined classical features using logistic regression model	6
Appendix Figure S7. Short linear motifs (SLiMs) identified to be enriched by SLiMFinder	7
Appendix Figure S8. Performance of ML methods on the random library	8
Appendix Figure S9. Sequence and structural analysis of TADs in complex with binding partners	9
Appendix Figure S10. Functional outcomes of the variants of the 13 different TAD sets in the design library	10
Appendix Figure S11. Distribution of the tolerance scores for the 13 different wild-type TAD sequences	11
Appendix Figure S12. Performance of ML methods on the design library	12
Appendix Figure S13. Performance of ML methods on the combined library	13

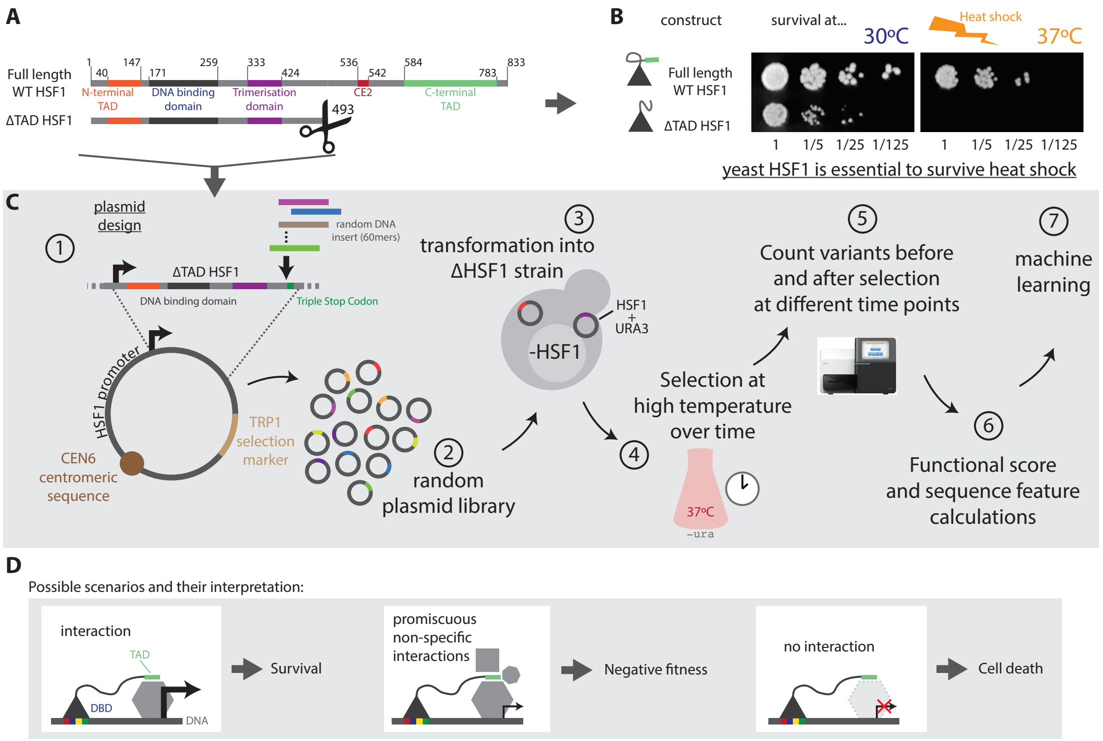

Appendix Figure S1. Details of the experimental design.

- **A.** Plasmid design. The inserted gene on the plasmid is a truncated version of the HSF1 gene, referred to as ΔTAD HSF1. The truncation is introduced following amino acid 493 and the constructs thereby include an N-terminal transactivation domain (N-TAD) necessary for survival at 30ºC, a DNA-binding domain with a helix-turn-helix fold (HTH) followed by a trimerisation domain that is involved in trimer formation, thereby increasing its binding affinity for DNA. In this respect, the ΔTAD HSF1 differs to the wild type only in that it misses the C-terminal transactivation domain (C-TAD) and the conserved element (CE2). The latter has been associated with repression of the activation capacity of the C-TAD[39]. Some portion of a flexible region linking the C-terminus to the rest of the protein was also removed.
- **B.** Yeast cells with ΔTAD HSF1 can survive under normal growth conditions, but are eradicated upon heat shock. Hence HSF1's C-TAD is an essential portion of the protein to survive heat shock.
- **C.** Adapting IDR-Screen to discover and learn the properties of sequences that function as TADs. To provide the possibility to rescue the high temperature growth phenotype, a set of restriction enzyme (RE) sites was introduced right at the truncation point (amino acid 493) in order to provide an integration site for a library of DNA fragments that can code for peptides, substituting the wild type C-TAD. Furthermore, native promoter was used in order to keep the expression of ΔTAD HSF1 near native levels. The plasmid contains standard features to enable genetic manipulation. The plasmid contains a positive selection marker (TRP1), which was used to ensure plasmid uptake. The plasmid contains a centromeric sequence (CEN6), which ensures that every transformed yeast cell will have a single copy of the plasmid, or at least have it at low copy number. At the 3' end of the construct, we inserted a Triple Stop Codon to ensure that translation would stop in case there are no stop codons in the variant sequence. The frame of translation for the majority of 60-mers was used to set the first of the Stop codons so that there would not be a consistent bias of amino acids at the end of the peptide once translated. The ΔHSF1 strain into which the plasmids are transformed contain a full size HSF1 on an additional URA3 plasmid, which in turn is shuffled out during the temperature selection in–trp, 5FOA medium.
- **D.** Possible outcomes during the screening and their interpretation. (left) The sequence mediates interaction with the transcriptional machinery, leading to its recruitment and cell survival. (middle) The sequence mediates promiscuous non-specific interactions that lead to ineffective recruitment of the transcriptional machinery and hence results in negative growth fitness. (right) The sequence mediates no interaction, leading to the inability to recruit the transcriptional machinery, thereby resulting in cell death.

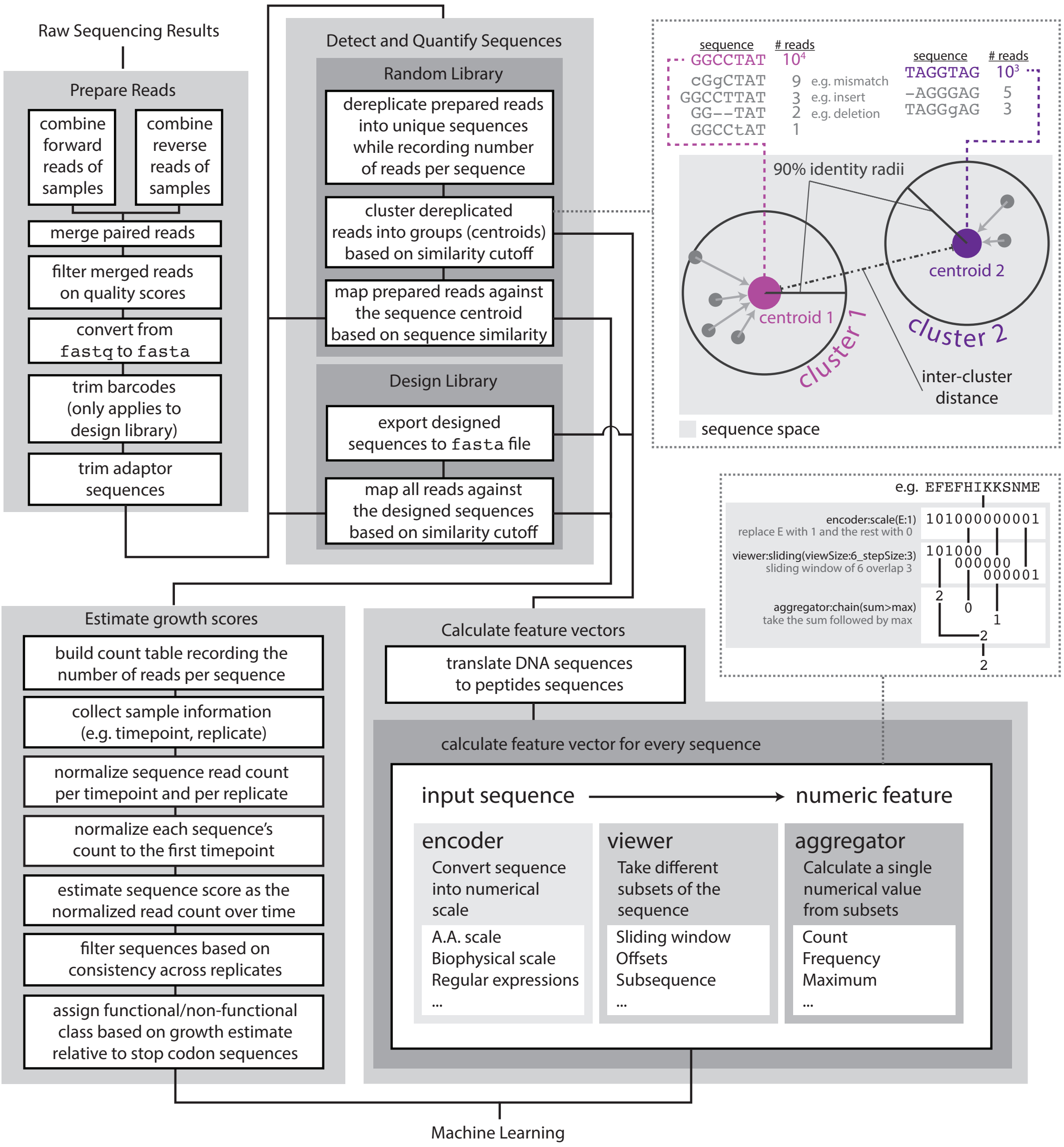

Appendix Figure S2. Flowchart of the next generation sequencing data processing pipeline and Machine Learning.

The computation part of IDR-Screen is divided into four stages: processing of reads, calculating scores to define functional and non-functional sequences, calculating features of the sequences in the library and performing machine learning. After the selection experiment, the raw read files are processed to be in a form where individual sequences can be counted to reflect their strength of selection, which is linked to the function. In parallel, for every sequence in the library, a range of descriptive features is calculated that describe them in numerical vectors. Finally, the features are used to learn to predict functional sequences in a Machine Learning step.

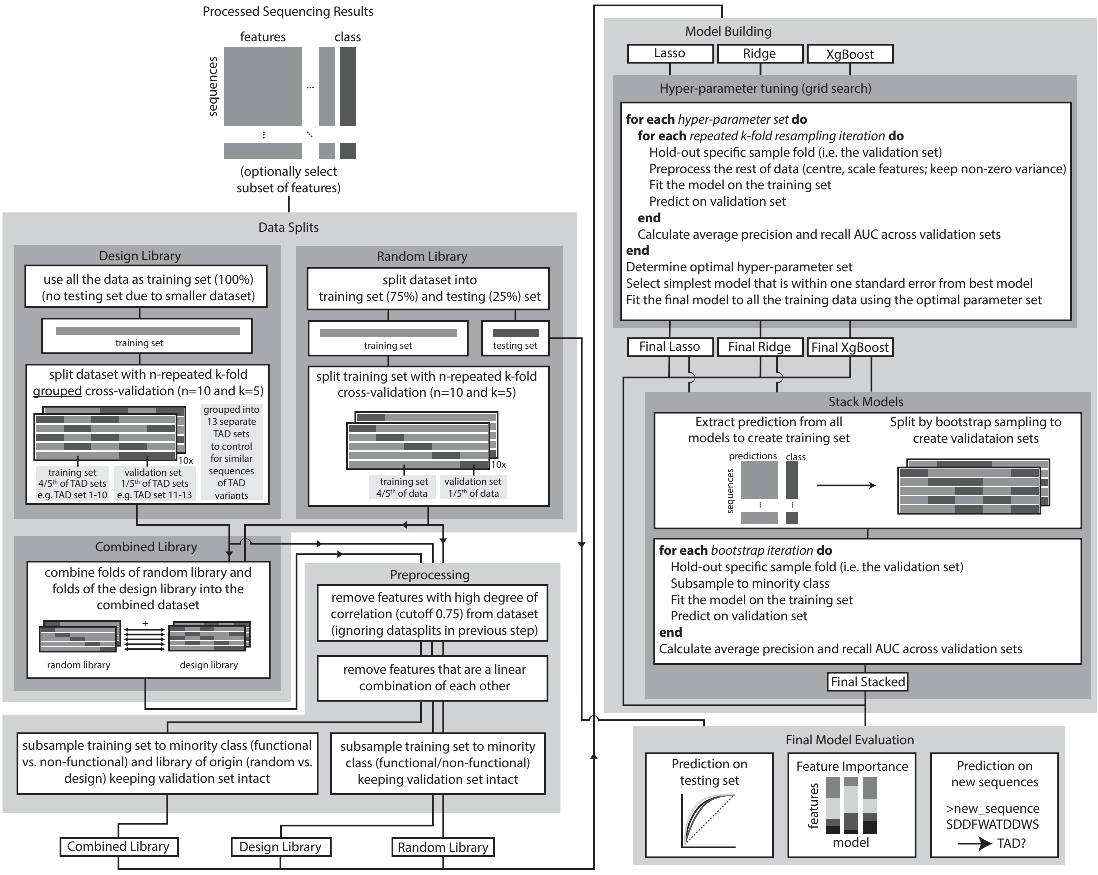

Appendix Figure S3. Machine learning procedure flowchart.

Flowchart describing the details of the machine learning procedure. Please see Materials and Methods for details.

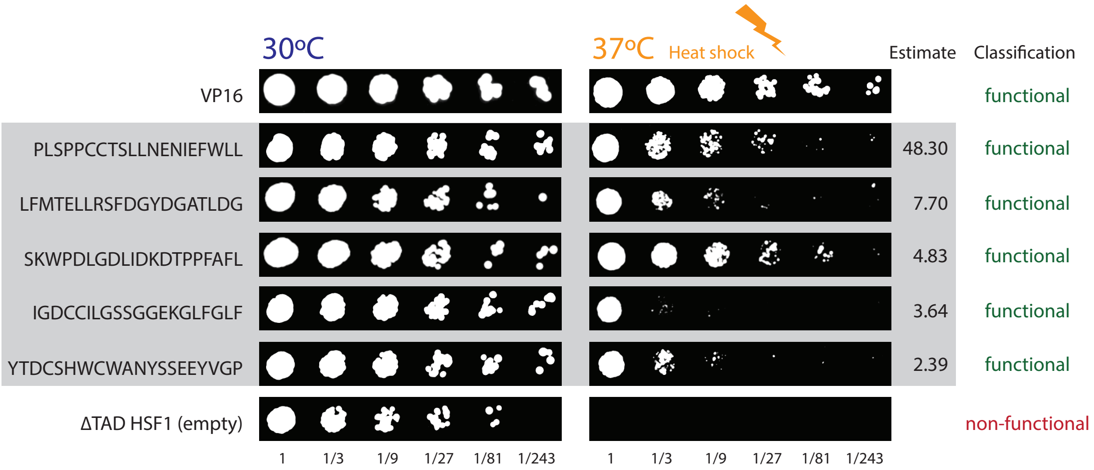

Appendix Figure S4. Spot dilution assay for sequences identified during the screen of the random library.

This was performed both at permissible temperature for ΔHSF1 strains to grow (30ºC) and at non-permissible heat shock temperatures of 37ºC. VP16 and ΔTAD-HSF1 strains are given as reference points. Growth estimates (in a.u.) and classification are shown on the right.

A

Precision-Recall AUC metric

feature	training	testing
length	0.0129	0.0117
pI	0.0291	0.0229
hydrophobicity	0.0135	0.0130
9 AA TAD	0.0163	0.0114
disorder	0.0114	0.0107
helicity	0.0128	0.0111

imbalance ratio: 0.0115

ROC AUC metric

feature	training	testing
length	0.5456	0.5141
pI	0.6544	0.6121
hydrophobicity	0.5644	0.5394
9 AA TAD	0.5614	0.5060
disorder	0.5272	0.4922
helicity	0.5246	0.5009

B length

Predicted non-functional, Observed non-functional

sequence	value	length
FNTSGPLSSLWSL	13	13
CCPAPMEGDFWFR	13	13
PRALVRQPCAS	11	11
VSERRSVQEKHR	12	12

Predicted non-functional, Observed functional

sequence	value	length
DPVSLAYRDFWDY	13	13
PTSSFAPWDLLM	12	12
CGIIQFNDLLYS	12	12
LRLWFDPFFYS	11	11

Predicted functional, Observed non-functional

sequence	value	length
PDSLNLFDFSRPWTALSPWW	20	20
MDMYQEPRRLPWTARRRECA	20	20
YCNDWGETHLNIRYWLGNQP	20	20
ERTPAQVHQIEQKSRFLRRI	20	20

Predicted functional, Observed functional

sequence	value	length
ASWCSDDPELEAEFSLLVRW	20	20
YYLTECQELDPTLWIPKCGP	20	20
DNNFLWASDTVSWLEEPLAN	20	20
CISPDMSIAYWEGWWSL	17	17

C pI

Predicted non-functional, Observed non-functional

sequence	value	length
MDMYQEPRRLPWTARRRECA	9.49	20
ERTPAQVHQIEQKSRFLRRI	11.54	20
IPQVVVVYIDHRKIRR	10.90	16
QISVHTLPATPKRQNRPPPS	12.01	20

Predicted non-functional, Observed functional

sequence	value	length
YVTRQFCIEVLPVARGFPRI	9.50	20
WRRASCSVQAMGSGLL	10.35	16
PWVIPRREDSTIQLLRP	9.94	17
PYMSFSAVHRASLMRTCRVN	10.77	20

Predicted functional, Observed non-functional

sequence	value	length
PDSLNLFDFSRPWTALSPWW	4.21	20
YCNDWGETHLNIRYWLGNQP	5.32	20
TCAPLFWQASQSQLRGEALI	5.66	20
FNTSGPLSSLWSL	5.52	13

Predicted functional, Observed functional

sequence	value	length
ASWCSDDPELEAEFSLLVRW	3.83	20
YYLTECQELDPTLWIPKCGP	4.14	20
DPVSLAYRDFWDY	3.93	13
DNNFLWASDTVSWLEEPLAN	3.43	20

D hydrophobicity

Predicted non-functional, Observed non-functional

sequence	value	length
MDMYQEPRRLPWTARRRECA	0.25	20
YCNDWGETHLNIRYWLGNQP	0.30	20
ERTPAQVHQIEQKSRFLRRI	0.25	20
GVGDYQSVTSFSQSSLNKI	0.26	19

Predicted non-functional, Observed functional

sequence	value	length
DPVSLAYRDFWDY	0.31	13
SIAEDTMDMWAGWYLPRYPA	0.30	20
GQPVSDTVVWDWGFTAQEEP	0.30	20
FGTQVHDDYSAMLGYTWAYT	0.25	20

Predicted functional, Observed non-functional

sequence	value	length
PDSLNLFDFSRPWTALSPWW	0.40	20
TCAPLFWQASQSQLRGEALI	0.35	20
FNTSGPLSSLWSL	0.38	13
RQFENSVSGILWGYLQELDL	0.40	20

Predicted functional, Observed functional

sequence	value	length
ASWCSDDPELEAEFSLLVRW	0.40	20
YYLTECQELDPTLWIPKCGP	0.35	20
DNNFLWASDTVSWLEEPLAN	0.35	20
CISPDMSIAYWEGWWSL	0.47	17

E disorder

Predicted non-functional, Observed non-functional

sequence	value	length
MDMYQEPRRLPWTARRRECA	0.63	20
ERTPAQVHQIEQKSRFLRRI	0.73	20
FNTSGPLSSLWSL	0.59	13
CCPAPMEGDFWFR	0.66	13

Predicted non-functional, Observed functional

sequence	value	length
PTSSFAPWDLLM	0.74	12
GQPVSDTVVWDWGFTAQEEP	0.77	20
RDKSAHPDSGDVLFLTLLSD	0.62	20
FQPASSAHGSILECLIVPEE	0.59	20

Predicted functional, Observed non-functional

sequence	value	length
PDSLNLFDFSRPWTALSPWW	0.35	20
YCNDWGETHLNIRYWLGNQP	0.36	20
TCAPLFWQASQSQLRGEALI	0.46	20
RQFENSVSGILWGYLQELDL	0.27	20

Predicted functional, Observed functional

sequence	value	length
ASWCSDDPELEAEFSLLVRW	0.47	20
YYLTECQELDPTLWIPKCGP	0.41	20
DPVSLAYRDFWDY	0.42	13
DNNFLWASDTVSWLEEPLAN	0.54	20

F helicity

Predicted non-functional, Observed non-functional

sequence	value	length
PDSLNLFDFSRPWTALSPWW	0.09	20
TCAPLFWQASQSQLRGEALI	0.26	20
FNTSGPLSSLWSL	0.13	13
RQFENSVSGILWGYLQELDL	0.29	20

Predicted non-functional, Observed functional

sequence	value	length
YYLTECQELDPTLWIPKCGP	0.03	20
DNNFLWASDTVSWLEEPLAN	0.17	20
LSWFFLADVENSDLDTEGNG	0.16	20
PTSSFAPWDLLM	0.17	12

Predicted functional, Observed non-functional

sequence	value	length
MDMYQEPRRLPWTARRRECA	0.69	20
YCNDWGETHLNIRYWLGNQP	1.41	20
ERTPAQVHQIEQKSRFLRRI	0.71	20
GVGDYQSVTSFSQSSLNKI	0.43	19

Predicted functional, Observed functional

sequence	value	length
ASWCSDDPELEAEFSLLVRW	1.63	20
DPVSLAYRDFWDY	0.64	13
CISPDMSIAYWEGWWSL	0.61	17
INGFYEPLRELIWEF	0.54	15

G 9 aa TAD

Predicted non-functional, Observed non-functional

sequence	value	length
PDSLNLFDFSRPWTALSPWW	0	20
MDMYQEPRRLPWTARRRECA	0	20
RQFENSVSGILWGYLQELDL	0	20
CCPAPMEGDFWFR	0	13

Predicted non-functional, Observed functional

sequence	value	length
YYLTECQELDPTLWIPKCGP	0	20
DNNFLWASDTVSWLEEPLAN	0	20
INGFYEPLRELIWEF	0	15
LSWFFLADVENSDLDTEGNG	0	20

Predicted functional, Observed non-functional

sequence	value	length
YCNDWGETHLNIRYWLGNQP	1	20
ERTPAQVHQIEQKSRFLRRI	1	20
TCAPLFWQASQSQLRGEALI	1	20
FNTSGPLSSLWSL	1	13

Predicted functional, Observed functional

sequence	value	length
ASWCSDDPELEAEFSLLVRW	2	20
DPVSLAYRDFWDY	2	13
CISPDMSIAYWEGWWSL	1	17
SIAEDTMDMWAGWYLPRYPA	2	20

Appendix Figure S5. Performance of classical features associated with TAD using logistic regression models.

- **A.** Precision-Recall AUC and ROC AUC of the different features traditionally associated with TADs (length, pI, hydrophobicity, disorder, helicity, and the presence of a 9 aa TAD motif). The imbalance ratio describes the value on which a random predictor would lie.
- **B-G.** The confusion matrices for the different baseline models are given along with the balanced accuracies and some example sequences. Example sequences by the baseline models using:
- **B.** length
- **C.** pI
- **D.** hydrophobicity
- **E.** disorder
- **F.** helicity
- **G.** The presence of a 9 aa TAD motif.

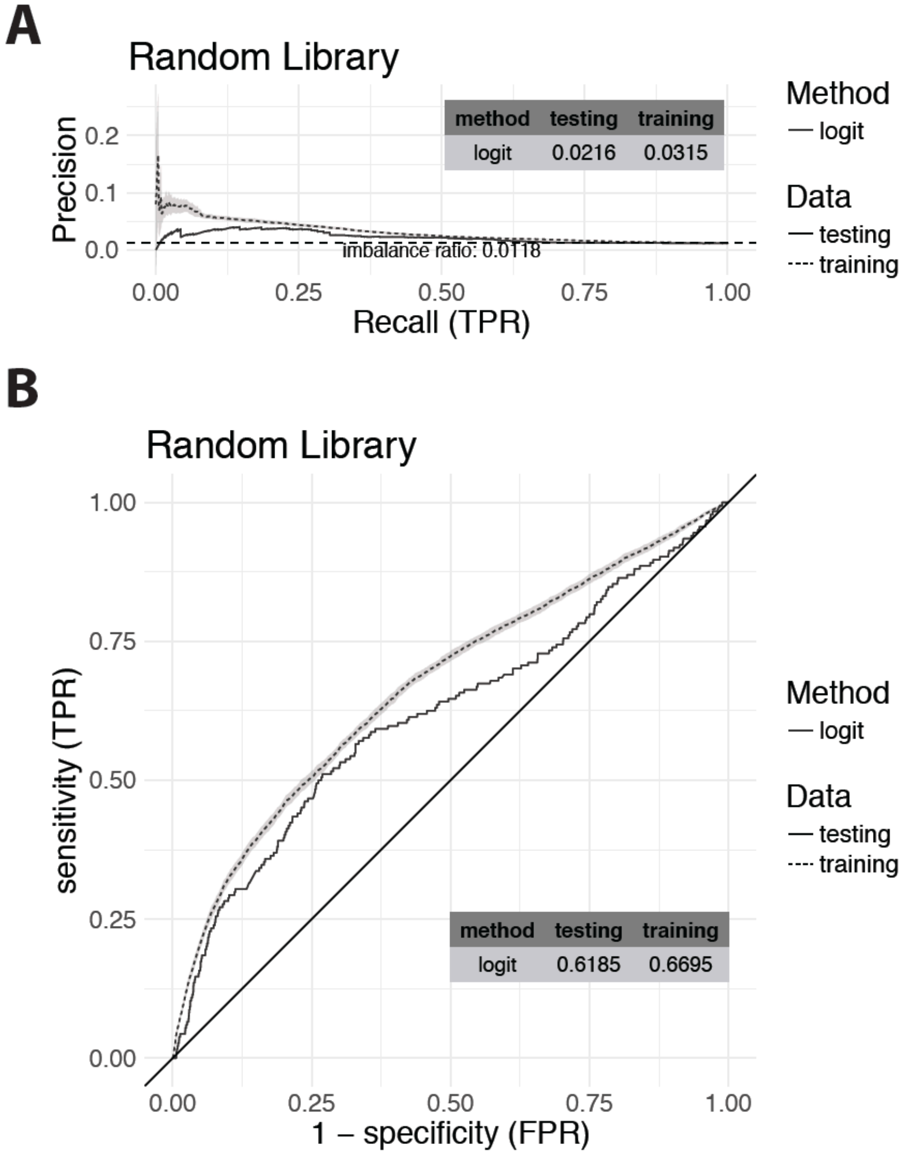

Appendix Figure S6. Performance of the combined classical features using logistic regression model.

- **A.** Precision recall curve of the logistic regression model, which considers the different features typically associated with TADs (length, pI, hydrophobicity, disorder, helicity, and the presence of a 9 aa TAD motif) together.
- **B.** ROC of the same model. AUC values are provided as inset. The imbalance ratio describes the value on which a random predictor would lie. The line-type represents the training and the testing dataset split respectively (see **Appendix Figure S3**). Error margins are shown for the training sets (light gray).

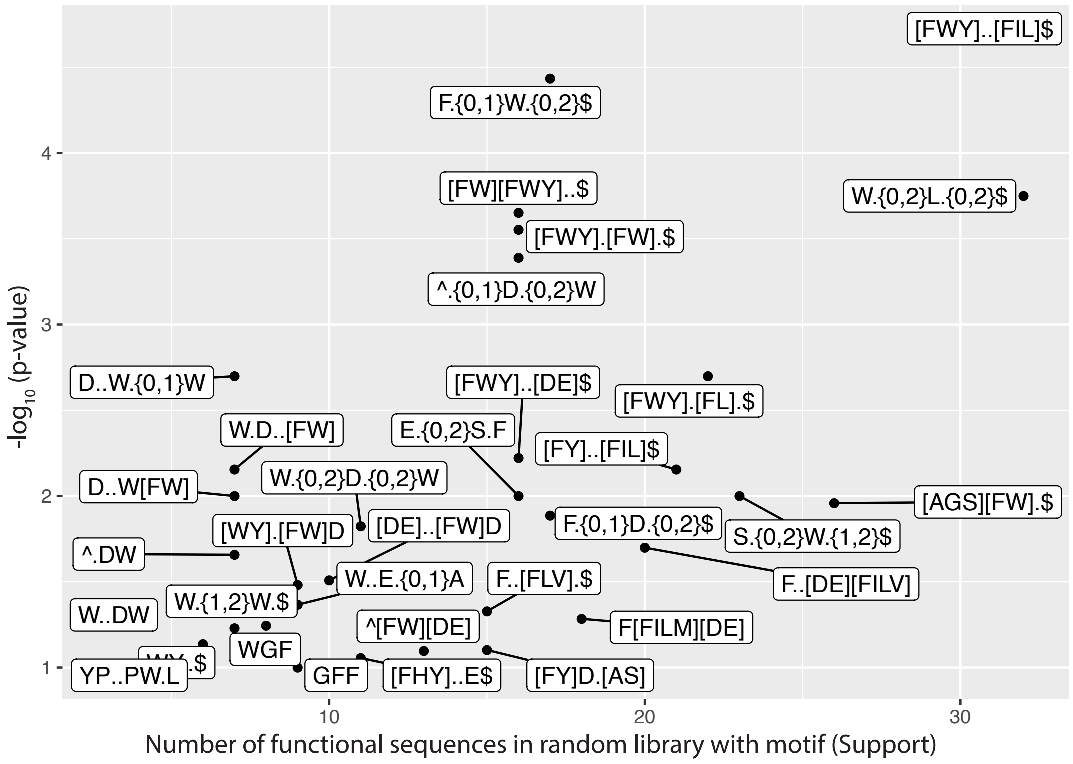

Appendix Figure S7. Short linear motifs (SLiMs) identified to be enriched by SLiMFinder.

The x-axis represents the number of times a motif was discovered in the sequence set of the random library (Support) and the y-axis represents the statistical significance, $-\log_{10}$ (p-value), of each motif. SLiMFinder can be accessed at http://www.slimsuite.unsw.edu.au/servers/slimfinder.php.

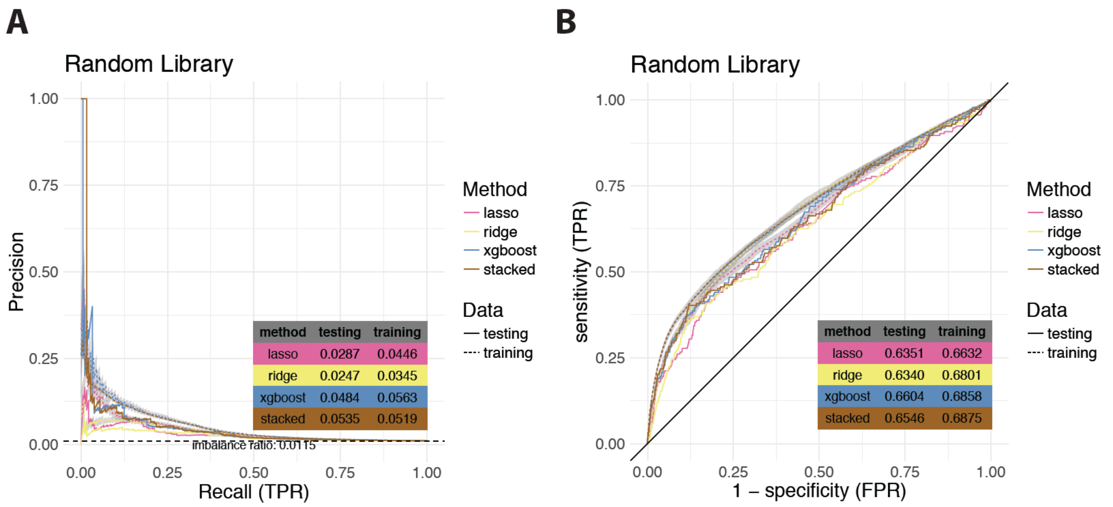

Appendix Figure S8. Performance of ML methods on the random library.

A. Precision recall curve and

B. ROC of the models trained with the random library dataset. The different colors represent the different methods used to train the models. The line-type represents the training and the testing dataset split respectively (see **Appendix Figure S3**). Values in the table represent the AUC. The imbalance ratio describes the value on which a random predictor would lie.

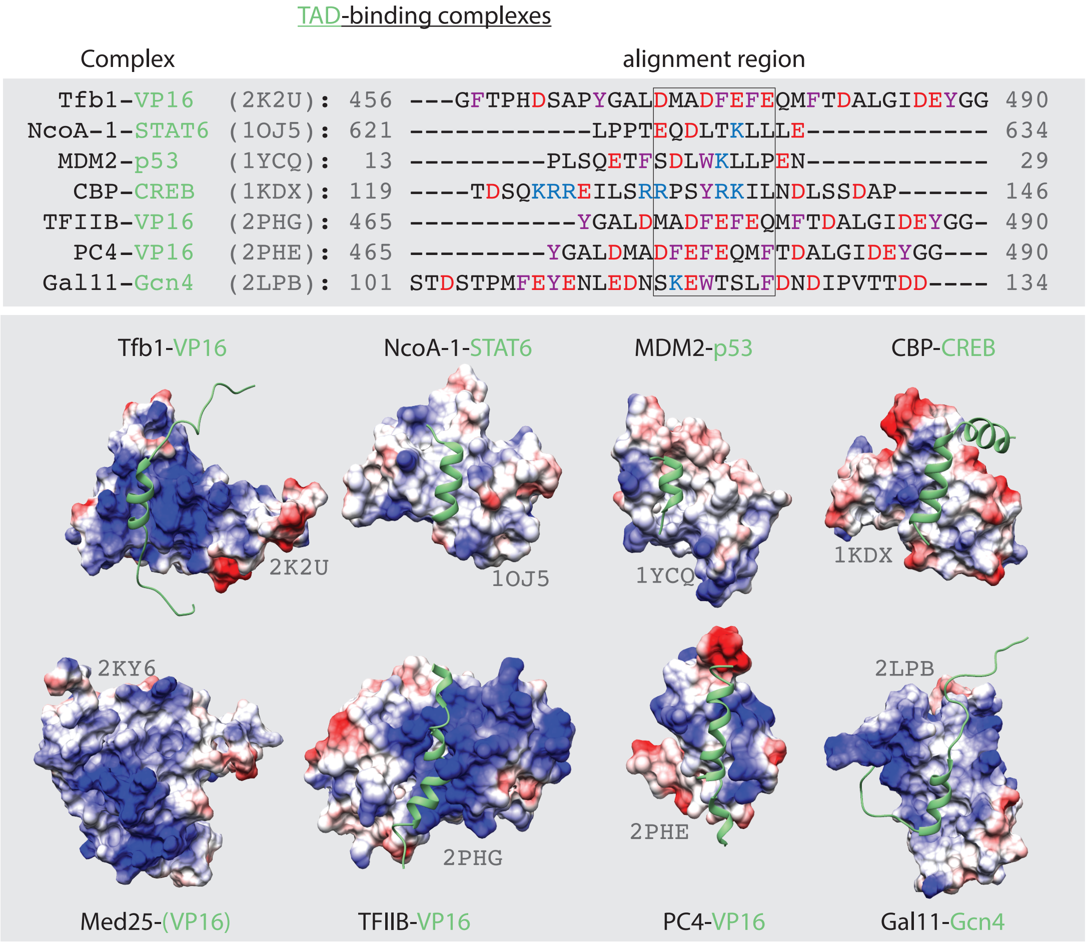

Appendix Figure S9. Sequence and structural analysis of TADs in complex with binding partners.

The structures of multiple complexes of transactivation domains (TADs) binding their respective partners is shown (bottom), along with an alignment of their sequences (top). The structures of the TAD interacting proteins were oriented based on the helical region of the respective TAD (green) that they contain (alignment region; grey box in top panel). The sequences of the TADs are provided in the panel above with acidic, aromatic and basic residues highlighted (red, purple and blue respectively). PDB IDs are provided near every complex structure. The position of the TADs in the respective factors hosting them is indicated in grey at the beginning and end of every sequence in the alignment. Within the alignment region, all activation domains host a large hydrophobic residue that consistently faces the interaction partner and that is buried. The surfaces of the TAD interacting domains were colored according to their electrostatic potential (blue, positively charged; red, negatively charged). The amino acid sequences of model acidic TADs (HSF_YEAST, HSF_KLULA, GCN4_YEAST, GAL4_YEAST, VP16_HHV1F and P53_HUMAN) were taken from the Uniprot database (http://www.uniprot.org/) and are shown in the alignment above.

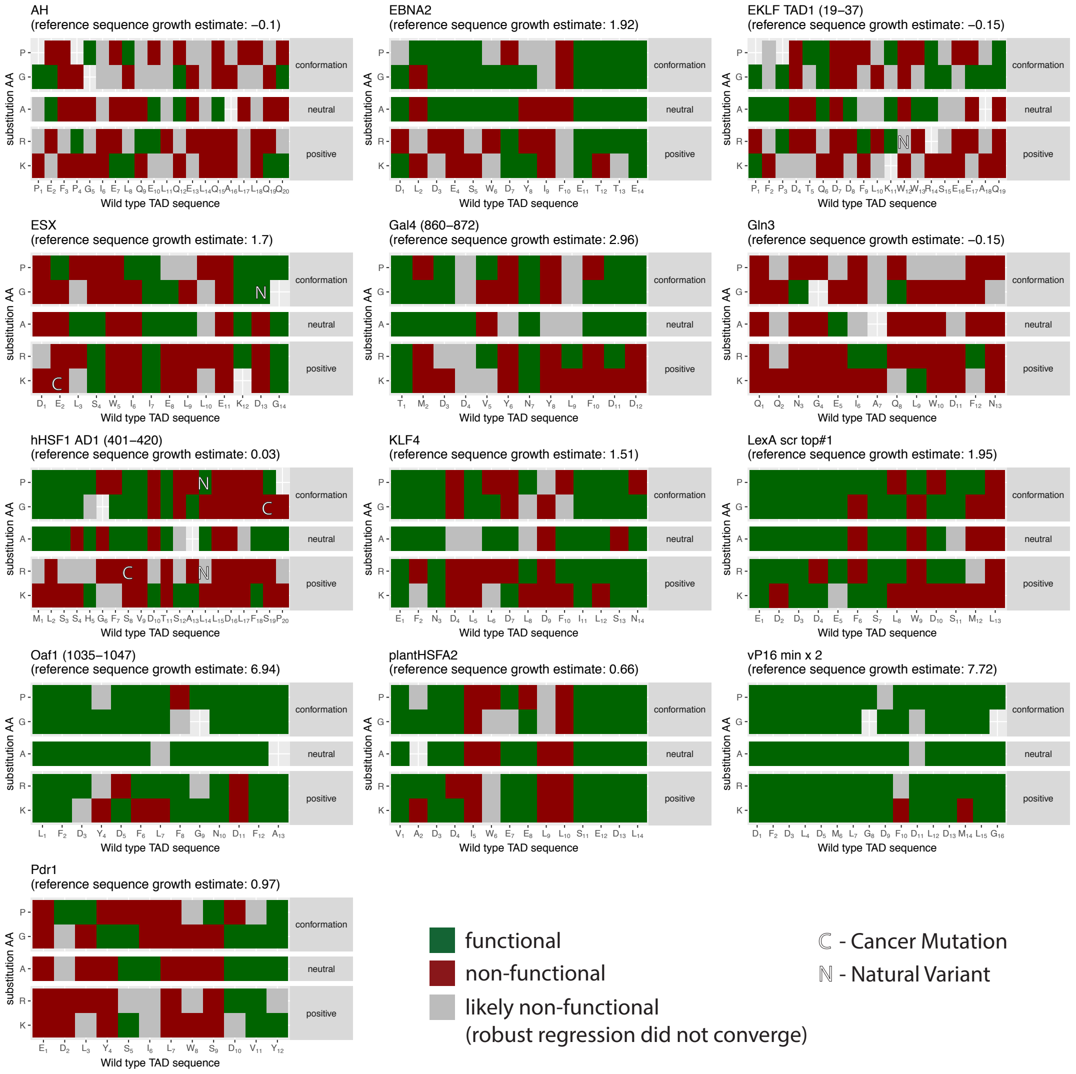

Appendix Figure S10. Functional outcomes of the variants of the 13 different TAD sets in the design library.

Tile plot of individual point mutations for the different variants of 13 WT TAD sequences. On the x-axis is the reference sequence of the WT TAD and on the y-axis is the amino acid that is used for the substitution. Mutations resulting in functional sequences are marked with green tiles and those that do not result in functional sequences are marked with a red tile. Disease mutations and natural variants in human proteins are marked with C and N respectively (**Methods** and **Table EV6**).

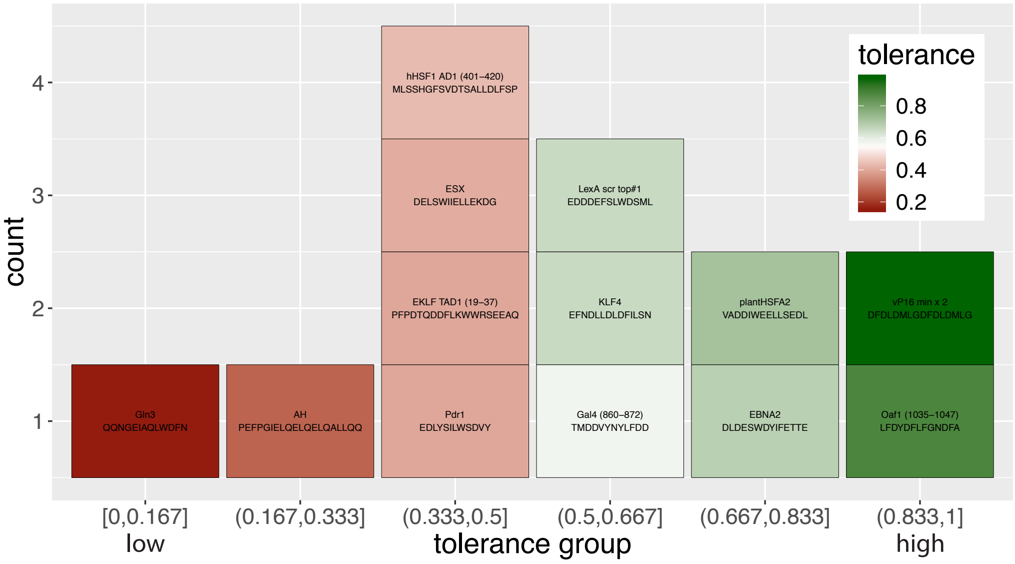

Appendix Figure S11. Distribution of the tolerance scores for the 13 different wild-type TAD sequences.

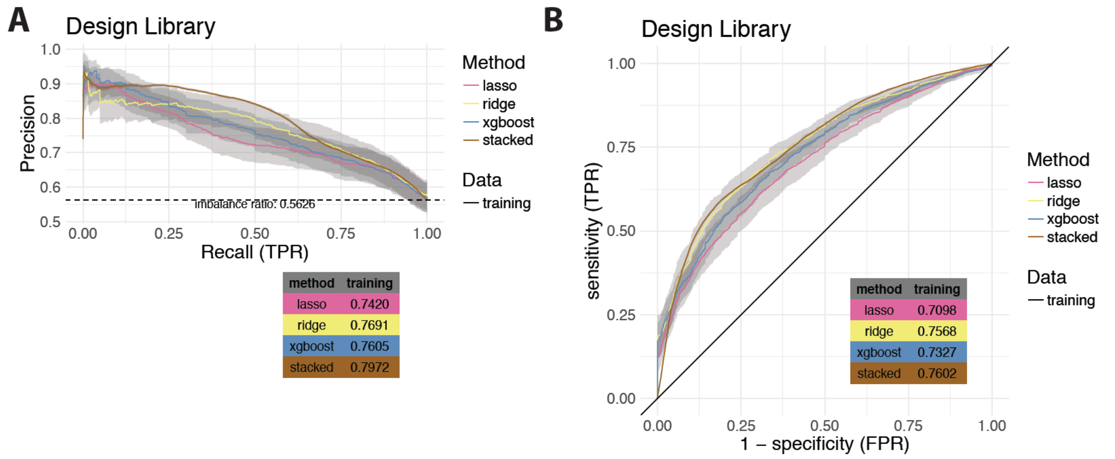

Appendix Figure S12. Performance of ML methods on the design library.

A. Precision recall curve

B. ROC of the models trained with the design library dataset. The different colors represent the different methods used to train the models. The line-type represents the training and the testing dataset split respectively (see **Appendix Figure S3**). Values in the table represent the AUC. The imbalance ratio describes the value on which a random predictor would lie.

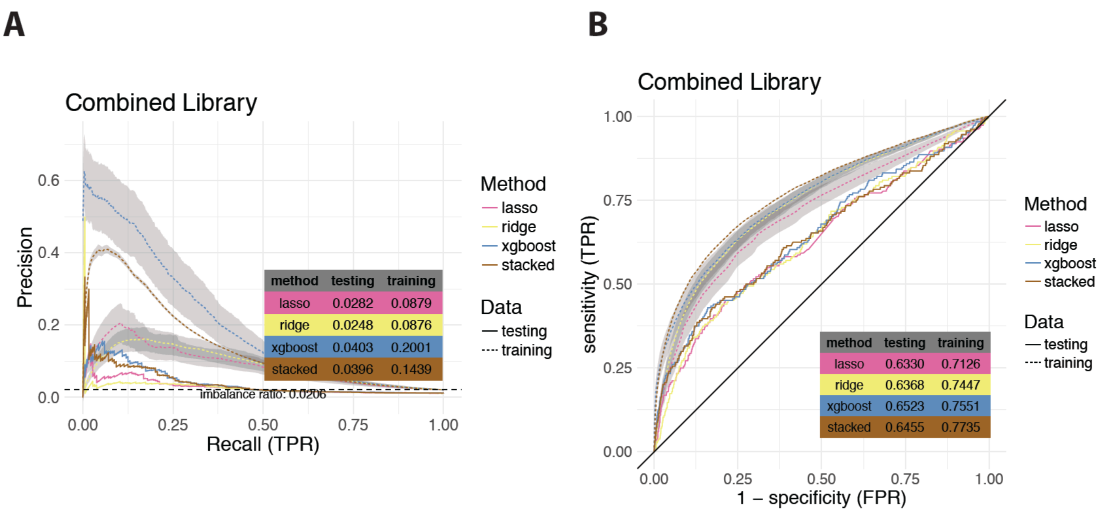

Appendix Figure S13. Performance of ML methods on the combined library.

- **A.** Precision recall curve. We note that the performance remains comparable to the models trained purely based on the random library.
- **B.** ROC of the models trained with the combined library dataset. The different colors represent the different methods used to train the models. The line-type represents the training and the testing dataset split respectively (see **Appendix Figure S3**). Values in the table represent the AUC. The imbalance ratio describes the value on which a random predictor would lie.
